# A Review of Computational Methods for Cervical Cells Segmentation and Abnormality Classification

**DOI:** 10.3390/ijms20205114

**Published:** 2019-10-15

**Authors:** Teresa Conceição, Cristiana Braga, Luís Rosado, Maria João M. Vasconcelos

**Affiliations:** Fraunhofer Portugal AICOS, 4200-135 Porto, Portugal

**Keywords:** cervical cancer, pap-smear, screening, machine learning, segmentation, classification, computer-aided diagnosis

## Abstract

Cervical cancer is the one of the most common cancers in women worldwide, affecting around 570,000 new patients each year. Although there have been great improvements over the years, current screening procedures can still suffer from long and tedious workflows and ambiguities. The increasing interest in the development of computer-aided solutions for cervical cancer screening is to aid with these common practical difficulties, which are especially frequent in the low-income countries where most deaths caused by cervical cancer occur. In this review, an overview of the disease and its current screening procedures is firstly introduced. Furthermore, an in-depth analysis of the most relevant computational methods available on the literature for cervical cells analysis is presented. Particularly, this work focuses on topics related to automated quality assessment, segmentation and classification, including an extensive literature review and respective critical discussion. Since the major goal of this timely review is to support the development of new automated tools that can facilitate cervical screening procedures, this work also provides some considerations regarding the next generation of computer-aided diagnosis systems and future research directions.

## 1. Introduction

Cervical cancer is the fourth most common cancer in women worldwide, and the second most frequent in low-income countries [1]. Globally, there are an estimated 570,000 new cases and 311,000 deaths from cervical cancer each year, 85% of them occurring in low and middle-income countries [2]. In 55 countries, it represents the form of cancer that kills the most women, generally in sub-Saharan Africa, Asia and Central and South America. The higher death rates in these countries are mainly caused by lack of effective prevention and equal access to early detection and treatment programs [3].

The increasing interest in the development of computer-aided diagnosis (CADx) systems for cervical cancer screening is closely related with the common practical difficulties experienced in these under-resourced health facilities, such as the shortage of specialized staff and equipment. Computer vision and machine learning approaches are often used in CADx systems to reduce the dependence of manual microscopic examination of cervical cytology smears, which is an exhaustive and time consuming activity, simultaneously requiring a considerable expertise of the cytotechnologist. During recent years, several computational approaches have been proposed to support cervical cancer screening. There is a wide rage of computer vision tasks that are highly relevant for this application area, such as: automated handling of smears variability; detection of artifacts; segmentation of individual cells and cell clusters; segmentation of nuclei and cytoplasm for each individual cell; and automated detection of abnormal changes in cell morphology.

Under the scope of this paper, various computer vision and machine learning approaches, already proposed in the literature, for the segmentation and classification of cells in microscopic images of cervical cytology smears were collected and reviewed. This timely review aims to support the increasing interest in the development of automated tools that can facilitate cervical screening procedures, especially in areas with limited access to quality healthcare services.

This document is structured into six sections. Section 1 corresponds to the Introduction and presents the motivation and objectives of this literature review. Section 2 gives an overview of cervical cancer disease in terms of cellular changes. Section 3 outlines the current cervical cancer screening strategies, with a major focus on cervical cytology. Section 4 gives a literature review of focus and adequacy assessment, segmentation and classification computational approaches used for the analysis of microscopic images from cervical cytology smears. Section 5 summarizes and gives a critical appreciation of the reviewed works. Finally, Section 6 provides the final remarks about the presented work.

## 2. Cervical Cancer Disease Characterization

Cervical cancer is a disease where malignant cells form and grow slowly in the tissues of the cervix, through an epithelial cell transformation. This transformation results in an epithelial dysplasia with the appearance of abnormal cells that is an early form of a precancerous lesion.

The major risk for the appearance of this cancer is to be persistently infected with cancer-causing Human papillomavirus (HPV) types, which are sexually transmitted, not only by penetration, but also by skin-to-skin contact of the genital areas [2,3,4]. HPV exists in more than 200 types, but they are short-lived, usually clearing up without any intervention within a few months after getting infected, and about 90% are spontaneously eliminated by the body within 2 years [2,3]. There are, at least, 13 known types of HPV that can persist and progress to cancer, named high-risk or oncogenic HPV types. HPV-16 and HPV-18 are high-risk types that cause 70% of cervical cancers and precancerous cervical lesions [2,3].

While infection with a high-risk HPV type is the underlying cause of almost all cases of cervical cancer, it is important to note that these infections do not always cause cancer [5]. Early forms of cervical cancer may not have signs or symptoms, but they can be detected through a regular Pap test, which is a procedure that consists of scrapping cells from the cervix to be looked at under a microscope [4,6,7]. If precancerous lesions are not treated, they can progress to cancer, in a process that can take about one to two decades [7]. When cervical cancer is already developed, it may include signs and symptoms such as vaginal bleeding, unusual vaginal discharge, pelvic pain or pain during sexual intercourse [6].

### Cervical Cancer Types

There are three categories of epithelial tumours of the cervix recognized by the World Health Organization (WHO): (i) squamous; (ii) glandular; and (iii) other epithelial tumours [8].

Squamous cell carcinomas are the flat, skin-like cells that cover the outer surface of the cervix [3,9]. As its name suggests, they are recognizably squamous, varying in either growth pattern or cytological morphology [8]. Around 70% to 80% of cervical cancers are squamous cell cancers [8,9], being predominant in most populations of HPV-16 and related types [10,11].

Adenocarcinoma cells start developing in the glandular cells that produce mucus, which are scattered along the inside of the passage that runs from the cervix to the womb (the endocervix) [3,9]. Adenocarcinomas are much less common than squamous cell carcinomas, accounting for about 15% of the cases [11]. Although both squamous cell carcinomas and adenocarcinomas have been associated with HPV infections, adenocarcinomas have been particularly associated with HPV-18 and related virus types.

Among other epithelial tumours, adenosquamous cancers are tumours that have both malignant squamous and malignant glandular cells (adenocarcinomas), making up about 33% of cervical carcinomas with glandular differentiation [9,12].

## 3. Cervical Cancer Screening Characterization

When screening women for cervical cancer, we might be looking for both pre-cancerous and cancerous lesions, so screenings can be conducted in women without any reported symptoms. In particular, with effective screening strategies, pre-cancerous lesions can be detected earlier and adequately treated, which is proven to prevent up to 80% of cervical cancers [2]. The main goal of this section is to provide a brief summary of key aspects related to cervical cancer screening, since we strongly believe that this in-depth medical and biological knowledge can greatly influence the design of CADx systems.

### 3.1. Screening Methods

The latest guidelines from World Health Organization (WHO) (2019), recommend three different types of screening tests [2]: (i) HPV testing for high-risk HPV types; (ii) visual inspection with acetic acid (VIA); and (iii) cervical cytology: conventional (Pap) test and liquid-based cytology (LBC).

**HPV Testing**: is similar to that performed to detect other DNA viruses, such as Adenovirus or Hepatitis B virus. It consists of a laboratory test that uses polymerase chain reaction (PCR) or hybrid capture to check DNA or RNA to find out if there is any cervical cancer-related HPV infection. If not a primary HPV testing, it can be performed as a consequence of finding abnormal cervical cells in a Pap test [6,13]. Testing for HPV (or genotyping) has demonstrated greater sensitivity for high-grade cervical intraepithelial neoplasia than cytology, and provides 60% to 70% greater protection against invasive cervical cancer [8]. These values support the fact that the literature has been pointing to HPV testing as the most accurate way of finding people at risk of developing abnormal cells in the cervix [8,13,14,15].

**VIA**: is a procedure that enables physicians to directly see cervical lesions using acetic acid on the cervix. While normal cervical tissues remain unaffected by the acetic acid, damaged tissues will become white after 1 to 2 min. Cervical lesions usually appear near the squamocolumnar junction [3]. When white areas are spotted, the physician can remove the damaged tissues using cryotherapy or other techniques. Although VIA yields high sensitivity, WHO does not recommend it for postmenopausal women, because it is more difficult to visualize the squamocoloumnar junction in older women, resulting in a poor performance of the test [3,13]. Moreover, VIA provides limited accuracy for detection of pre-cancerous lesions [16]. In effect, a randomized controlled trial in India that had a significant decrease in cervical cancer in women aged 30–39 years old, reports having no similar results in case of older ages [13]. It is, therefore, an attractive alternative to other screening methods in low-resource settings, but with poor intervention for women of older ages [17].

**Cervical Cytology**: has been a standard method for cervical cancer screening for many years, linked to drastic reductions of mortality rate in many countries of the world. Cervical cytology has reduced the incidence of cervical cancer by 60% to 90% and the death rate by 90% [8]. Currently, there are two types of cervical cytology tests being performed: (i) conventional Papanicolaou smear (CPS); and (ii) liquid-based cytology (LBC). While LBC is more popular in developed countries, CPS is more practiced in low resource settings [13]. In fact, many researches have been conducted to evaluate and compare the efficacy of LBC with CPS as a screening tool for cervical cancer. Results lead to the conclusion that LBC samples provide higher quality samples by offering better clarity and uniform spread of smears, which reduces the number of unsatisfactory cases and requires less time for screening [18,19,20]. This has even more impact in postmenopausal women, where menopause-induced anatomical and hormonal changes make screening particularly challenging [19]. Although LBC is attributed with superior quality with respect to the decreased number of unsatisfactory smears, some works have concluded that it does not offer significant differences in the diagnostic performance, which, considering the economic implications of LBC, makes CPS more feasible for settings where LBC is not affordable [21,22]. Despite the undeniable contribution that cervical cytology has had in decreasing cervical cancer-related deaths, cervical cytology has significant limitations including: being a morphological method, thus relying on the subjective interpretation by well-trained cytotechnologists; not optimal sensitivity (around 50%); and a high proportion of borderline results, leading to an excessive number of referrals for colposcopy [8,23,24].

The type of screening methods applied can differ across countries (single or co-testing) as well as the target ages, depending on the recommendations established or resources available [17,24,25].

### 3.2. Classification Systems

Existing classification systems for classifying cancerous or pre-cancerous lesions of the cervix may be based either on cytology or histology. Additionally, they can have different reporting and clinical purposes (see Table 1). Since the main driving force of our work is to support the development CADx systems that can facilitate screening procedures, we will only give a brief explanation of the Bethesda system (TBS).

#### The Bethesda System

TBS is a cytological classification that provides a standard set of terms to describe abnormal findings from CPS and LBC, which reports both sample adequability and cytology results [28].

**Figure 1:**
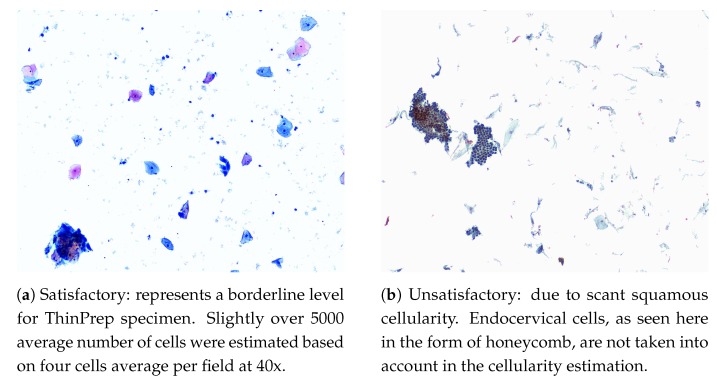
Satisfactory (**a**) and unsatisfactory (**b**) LBC preparations. From: Nayar, R.; Wilbur, D. *The Bethesda System for Reporting Cervical Cytology: Definitions, Criteria, and Explanatory Notes*, 3rd ed.; Springer International Publishing, 2015 [26] and reproduced with permission of Springer.

Several factors can hinder the interpretation of a certain specimen such as technical problems in the slide preparation, low cellularity, or obscuring factors like the excessive presence of blood, inflammations, bacteria or lubricant. Therefore, specimen quality assessment and its reasoning are an important step in reducing the number of false negative (FN) diagnosis and are often reported during manual screening. In order to decrease subjectivity and uniformize this analysis, laboratories are recommended to follow guidelines and minimum criteria [26]:

- **Cellularity**: a minimum of 5000 squamous cells on LBC and 8000–12,000 on CPS (Endocervical cells are not counted for this purpose). Examples of cellularity assessment are given in Figure 1.
- **Obscuring Factors**: unsatisfactory if more than 75% of the sample is obscured by blood, inflammatory cells, exudates or other artifacts.
- **Evidence of Transformation Zone**: 10 well-preserved endocervical cells or squamous metaplastic cells. Although this is an optional adequacy criterion, it is often included in reports.

If the sample is unsatisfactory for analysis, this provides the reason for inadequability and no cytology result is reported. If the sample is satisfactory for analysis, the main result may be one of two: either the cytology has an abnormal result, meaning that epithelial cells look abnormal, or the cytology is normal, meaning that no malignant lesions or epithelial abnormalities were found. Besides the absence of any abnormal cells, a negative result may also include benign findings, such as certain infections or inflammation [7].

Abnormal epithelial cells can be either atypical squamous cells (ASC) or atypical glandular cells (AGC), each cell type having a different grading system. Following, we present the different possible cytology results for ASC and AGC separately, listed in ascending order of harmfulness. In particular, a brief explanation of the expected visual changes that characterize each type is given (e.g., morphology, color, texture, etc.), since this established medical and biological knowledge can greatly influence the design of CADx systems for abnormal cytology screening.

**Figure 2:**
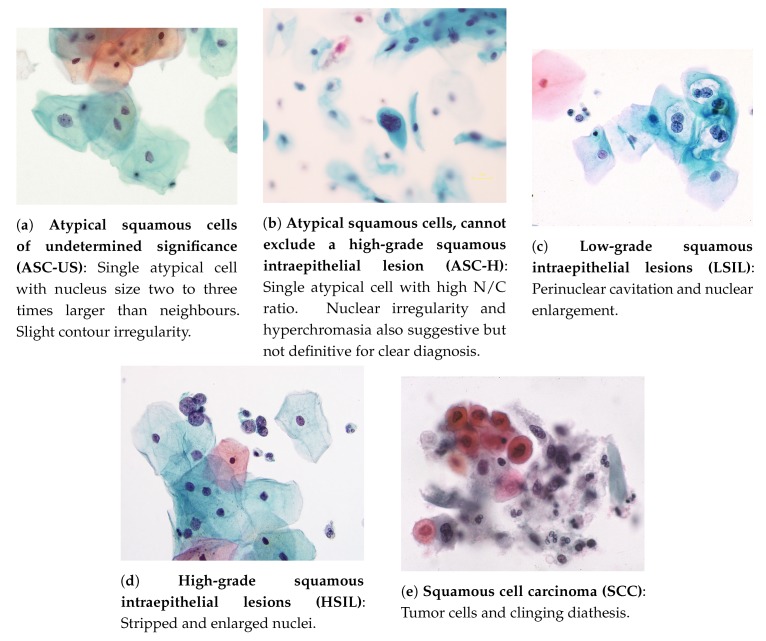
Atypical squamous cells on liquid-based cytology (LBC). From: Nayar, R.; Wilbur, D. *The Bethesda System for Reporting Cervical Cytology: Definitions, Criteria, and Explanatory Notes*, 3rd ed.; Springer International Publishing, 2015 [26] and reproduced with permission of Springer.

**ASC** comprises three essential features of analysis: (i) squamous differentiation; (ii) increased nuclear to cytoplasmic ratio; and (iii) minimal nuclear changes [26]. The following types can occur:

- *Atypical squamous cells of undetermined significance* (**ASC-US**): these cells are suggestive of low-grade squamous intraepithelial lesions (LSILs) and present a nucleus of approximately 2.5 to three times the area of a normal intermediate squamous cell nucleus (approximately 35 mm${}^{2}$) or twice the size of a squamous metaplastic cell nucleus (approximately 50 μm${}^{2}$) [26]. Example in Figure 2a.
- *Atypical squamous cells, cannot exclude a high-grade squamous intraepithelial lesion* (**ASC-H**): An interpretation of ASC-H is appropriate when atypical cells are undoubtedly present, but a clear distinction between high-grade squamous intraepithelial lesions (HSILs) or carcinoma is not viable. Example in Figure 2b.
- *Low-grade squamous intraepithelial lesions* (**LSILs**): to interpret a cell as a LSIL, nuclear abnormalities must be found. Characteristics of LSILs usually include nuclear enlargement, hyperchromasia (may be less evident in liquid-based samples), overall large cell size, “smudged” nuclear chromatin, well-defined cytoplasm, and multinucleation. Additional features of LSILs may, but are not required to, include perinuclear cavitation, a sharply defined perinuclear cavity, or condensation of cytoplasm around the periphery [26]. Example in Figure 2c.
- *High-grade squamous intraepithelial lesions* (**HSILs**): refers to cervical abnormalities that have a high likelihood of progressing to cancer if not treated [3]. The cells of HSILs are smaller than LSILs, showing less cytoplasmic maturity (see image below), and often contain quite small basal-type cells. Example in Figure 2d.
- *Squamous cell carcinoma* (**SCC**): the most prevalent malignant neoplasm of the uterine cervix, being defined as an invasive epithelial tumor composed of squamous cells of varying degrees of differentiation [29]. Commonly, a carcinoma appears as an isolated single cell, having notorious variations both in cellular size, shape, nucleus, and with possible irregular membranes [26]. Example in Figure 2e.

**Figure 3:**
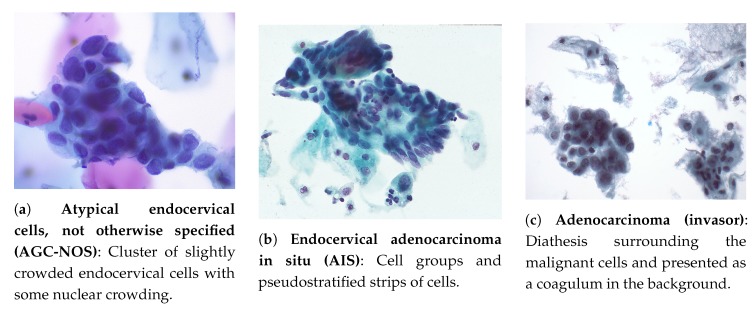
Atypical glandular cells on LBC. From: Nayar, R.; Wilbur, D. *The Bethesda System for Reporting Cervical Cytology: Definitions, Criteria, and Explanatory Notes*, 3rd ed.; Springer International Publishing, 2015 [26] and reproduced with permission of Springer.

**AGC** should be classified according to their site of origin, i.e., instead of only qualifying a lesion as glandular dysplasia, the qualification should include whether if endocervical or endometrial. It can be divided in four different types [26]:

- *Atypical endocervical cells, not otherwise specified* (**AGC-NOS**): occurrence in sheets and strips with some cell crowding, nuclear overlap, and/or pseudo stratification, nuclear enlargement, up to three to five times the area of normal endocervical nuclei, variation in nuclear size and shape, mild nuclear hyperchromasia, cell groups with rosettes (gland formations) or feathering (second image, second column) or small groups, usually five to ten cells per group (third image, second column) [26]. Example in Figure 3a.
- *Atypical glandular cells, favor neoplastic*: cell morphology practically suggests an interpretation of endocervical adenocarcinoma in situ or invasive carcinoma, but is not likewise enough to classify it that way. The criteria comprise the occurrence of abnormal cells in sheets and strips with nuclear crowding, overlap and/or pseudo stratification, rare cell groups with rosettes or feathering, among other characteristics [26].
- *Endocervical adenocarcinoma in situ* (**AIS**): represents for glandular abnormalities the same as HSILs to squamous cells and is considered the precursor of invasive endocervical adenocarcinoma. It consists of a non-invasive high-grade endocervical glandular lesion, characterized by nuclear enlargement, hyperchromasia, chromatin abnormality, pseudo-stratification, and mitotic activity [26]. Example in Figure 3b.
- *Adenocarcinoma (invasor)*: cytologic criteria matches those identified for AIS, but may contemplate additional signs of invasion [26]: (i) abundant abnormal cells, typically with columnar configuration; (ii) enlarged, pleomorphic nuclei; (iii) irregular chromatin distribution, chromatin clearing, and nuclear membrane irregularities; (iv) single cells, two-dimensional sheets or three-dimensional clusters, and syncytial aggregates; (v) cytoplasm is usually finely vacuolated; (vi) necrotic tumor diathesis (Tumor diathesis is a host response to tissue destruction by infiltrative growth of cancer [30], consisting of granular proteinaceous precipitates on slide surface of cytologic smears [31]) is common. Example in Figure 3c.

### 3.3. Datasets

**Figure 4:**
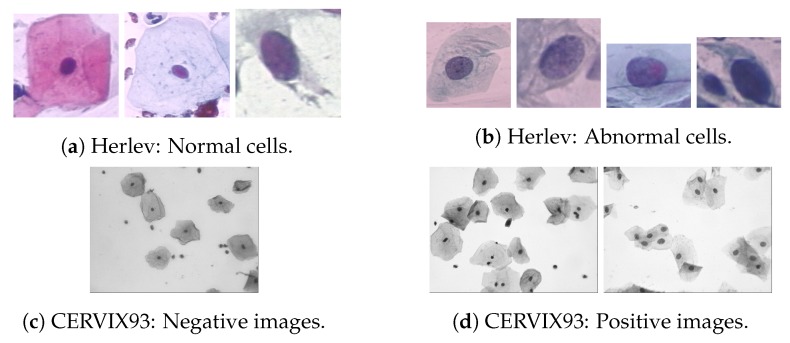
Sample images and corresponding classification of Herlev and CERVIX93 datasets.

**Figure 5:**
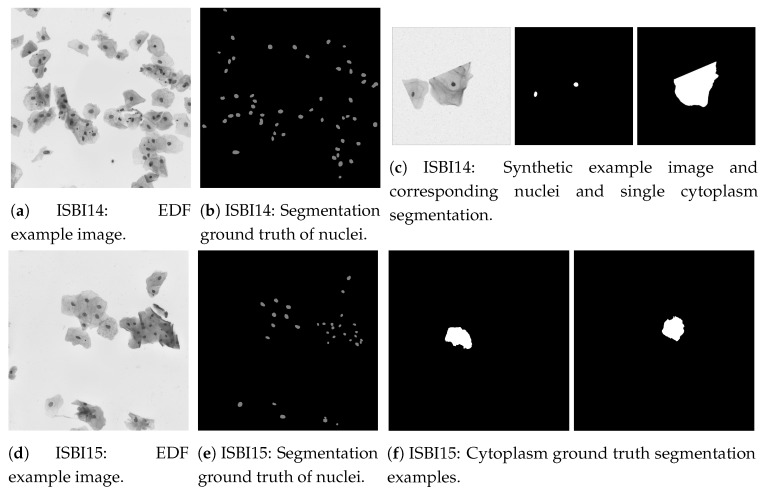
Sample images and corresponding segmentation masks of ISBI14 and ISBI15 datasets.

Computational-aided diagnosis applications require accurate and precise methods that can only be developed and validated using considerable amounts of labeled data. A lot of literature on cervical cancer screening and diagnosis is based on individual collected data. Although often meticulously authenticated, the number of samples is not usually adequate for large scale validation purposes. Furthermore, a great part of research works relies on replication and comparison which can only be done by having access to a proper baseline benchmark. Table 2 highlights the existing publicly available datasets while providing a short description of each, with examples being provided in Figure 4 and Figure 5.

### 3.4. Computer-Aided Commercial Systems for Cervical Cancer Screening

A number of commercial computer-aided systems have been developed to screen abnormal cervical cells in a semi-automatic (PAPNET) and automatic way (AutoPap 300, FocalPoint™, and ThinPrep Imaging System) [39].

The first devices, PAPNET and AutoPap 300, received the approval for re-screening of previously manually screened conventional smears by the United States Food and Drug Administration (FDA) in the 1990s. These systems have been proved to reduce the burdens of screening and increase productivity [40]. Comparative trials indicate that PAPNET selected true negative slides more accurately than conventional screening but there was no significant difference in the false negative rate, contrarily to AutoPap which claimed an increased sensitivity [39,40]. Despite promising results, PAPNET failed to demonstrate its cost-efficiency and was discontinued.

Regarding the AutoPap system, it was later approved for primary screening and renamed as FocalPoint™, being able to additional cope with LBC (BD SurePath preparations). The *Hologic Company* also developed a new system to be integrated with ThinPrep slides called ThinPrep Imaging System (TIS), which is not compatible with conventional Pap smears.

Thus, FocalPoint™ and TIS, are currently the only two commercially available FDA-approved automated screening systems that combine computer imaging technology with human interpretive expertise. Both scan slides at varying objective levels, applying several image processing and classification algorithms in order to select the fields of view (FOVs) with highest likelihood of having an abnormality. The selected FOVs are then electronically marked and showed in integrated workstations to the working staff. BD FocalPoint GS Imaging System ranks each slide and categorizes it into: review; no further review (NFR) (25% least likely to contain any abnormality); process review (technical problems or specimen inadequacy); and quality control review (selects 15% of the negative cases for a new full re-screen). The NFR slides can be flagged as negative and archived without human intervention. Contrarily, TIS selects the 22 higher-risk FOVs but does not assign scores to the whole slide, being unable to rank or directly archive slides without needing further human intervention or to select the most appropriate slides for quality control.

Overall, the systems’ interactivity improved cytotechnologists’ job satisfaction and productivity [40,41,42,43]. A mitigation of screening time by 42% (mean) (*p* < 0.001) was reported for TIS [43]. Additionally, unsatisfactory specimen detection rates were halved, which imply that image-assisted screening may have a better ability to identify abnormalities in samples with lower squamous cell cellularity [44,45]. Regarding the actual detection of abnormal cells, several studies authenticated these systems, reporting either equivalent or higher sensitivity [41,46,47]. However, other comparison commissioned by the Health Technology Assessment on LBC only, assessed a reduced sensitivity when compared to manual readings [46]. This suggests that despite irrefutable productivity gains, it is still uncertain to what extent can these devices be used as a primary screening tool.

## 4. Literature Review on Computational Approaches for Cervical Cytology

The ultimate goal of any CADx systems designed for screening purposes is to effectively support the detection of the highest number of potentially diseased patients. In such cases, it is of extreme importance not to miss any positive tests (zero false negatives), but achieving a good trade-off between high sensitivity and high specificity it is, for most automated systems, a particularly challenging task. Ultimately, automated screening can and should improve on manual testing in the following ways: (i) increase sensitivity and specificity of manual screening; (ii) decrease cytotechnologist workload; (iii) reduce screening programs cost; and (iv) reduce the probability of the disease incidence and mortality rate. Computerized cervical cells systems are usually based in one of the following three approaches [48]:

**The rare event approach (RE)**: The most commonly used systems are image analysis-based, scanning slide preparations while performing segmentation, feature extraction and classification of each individual cell. By comparing the number of suspicious cells with a pre-defined threshold, conclusions can be drawn regarding specimen classification. If enough evidence of abnormality is found, the scan does not need to be further analyzed, scanning can be stopped and time can be saved. Still, when it comes to normal samples, the least controversial approach is to scan the whole specimen before calling it normal and since potential (pre-)malignant cells are rare, this is not very time efficient.

**Malignancy associated changes (MAC)**: To deal with some of the disadvantages of the RE approach, such as problems in defining optimal thresholds or time efficiency, small chromatin texture changes at large neighborhoods can be assessed as they are often associated with specimen abnormality [48,49]. Because single cell chromatin texture has not been found to be enough evidence on abnormality, statistical analysis is made on a small population rather than on individual cells. Assuming these changes can be reliably detected, a complete scan is not required (data from around 500 cells is typically enough to characterize the whole chromatin distribution). In spite of presenting some advantages, MAC alone has still not been proven to provide highly satisfactory sensitivity [48].

**DNA ploidy approach**: The last and least common approach is to analyze nucleus DNA ploidy distribution in a stoichiometric stain, considering that normal cells entangle a diploid distribution as opposing to malignant cells whose distribution is aneuploid. It integrates optical density of all the nuclei, thus requiring very consistent and controlled procedures regarding staining, illumination and calibration. Furthermore, only individual, free-lying cells should be examined, so segmentation and artefact rejection are a crucial point. Studies on this approach state DNA ploidy as a potential attractive and reliable technology for countries lacking availability of skilled cytotechnologists due to the minimal training requirements and cost-effectiveness [50,51].

The general and most accepted approach is RE, but while MAC and DNA Ploidy are yet to be proven sufficiently reliable when used individually, some studies found that a hybrid combinational approach increases the overall system accuracy [52].

Artificial intelligence (AI)-powered solutions for cervical cancer screening use several computer vision and/or machine learning algorithms in order to identify and classify the extracted cells from the collected samples.

The previously depicted smear variability and presence of artefacts appear as a challenge to the implementation of these algorithms, which past and current research have been focusing on. The automated screening problem can be divided into four sub-problems which serve as the main basis for the following sub-sections: Focus Assessment, Adequacy Assessment, Segmentation and Classification. Device requirements and correct focusing are essential for a good quality acquisition system. Furthermore, morphological smear adequacy is also an important factor in order to ensure that the analysed sample is representative and can reliably be used to infer the final diagnosis. As a third step, segmentation deals with the proper identification of individual cells and/or cell clusters as well as proper cell part separation (nuclei and cytoplasm). Finally, the main goal of classification is to detect abnormal changes in the segmented cells morphology and diagnose the corresponding smear.

### 4.1. Focus Assessment

Shape and colour nuclei variations can usually be detected around the micron or sub-micron level [48]. Thus, accurate and reliable characterization requires high level microscopic magnification while maintaining an appropriate image quality. Additionally, cells in test slides are often spread in a multi-layer manner, especially for conventional preparations, hence needing different focus levels for proper digital representation. In order to accomplish this for an automated microscope without losing image quality, powerful auto-focusing techniques need to be employed. These work by mechanically finding the optimal z-axis position/focal plane for a given field of view obtained through the maximum value of a focusing function. In automated microscopy, due to time constraints, a good searching strategy passes by, first performing a large step coarse search and refines it later, only when significant differences between consecutive images are found [53].

In a study by Zhang et al. [54], different focusing functions were tested for a cost-effective cervical screening technique for developing countries, and a Gaussian filter with a common satisfaction degree of 98.2% was chosen. Focusing algorithms were also compared in a study by Liu et al. [55], in which a variance algorithm had the best overall performance for both blood and Pap smears. All in all, the best auto-focusing algorithm is application specific and should be selected according not only to accuracy error but also computational costs. Alternatively, the extended depth field (EDF) algorithm [35] has also been used recently for Pap smear slides [33,34]. Multi-layer cervical cell volumes at different levels of focus are stacked and combined in a single multi-focus image, where all of the objects in that image appear to be in focus. While this approach has the advantage of avoiding the strict processing time constraints required on algorithms that evaluate focus in real-time, on the other hand it requires much more data storage.

### 4.2. Adequacy Assessment

There is only one commercially available system, FocalPoint™, that provides feedback about adequacy assessment as it was previously mentioned in Section 3.4. However, it simply classifies a given slide into “process review” due to specimen inadequacy or other technical problems.

Several cell counting software tools currently available can be used for cellularity estimation [56,57,58,59]. Nevertheless, one should use them with caution as they were built to serve a more general purpose and not specifically designed to work with cervical cells. As an illustrative example, these tools do not distinguish squamous from endocervical cells, and the last type of cells should not be accounted for cellularity estimation purposes. Furthermore, there is not a considerable amount of research done on artefact rejection. This is by essence a difficult topic to address given the variability of objects or corruptions that can be present, and consequently an essential step to guarantee a reliable classification without having too many false positives.

Regarding methodologies in the literature for adequacy assessment of cervical cytology images, only a few works can be found, and mostly proposed as intermediate steps for segmentation and/or classification purposes [48]. Most research has been concentrated on distinguishing nuclei from inflammatory cells, extracting meaningful features and classifying each cell [60,61,62]. In terms of more general approaches for artifact rejection, Zhang et al. and Malm et al. [54,63] propose iterative filtering to remove increasingly complex cases. By operating in a sequential fashion, computational power is reduced since more complex analysis is only done in later phases, when most of the debris has already been discarded. Other implementations suggest the use of a support vector machine (SVM) classifier [64,65] or an fuzzy c-means [65] to remove unwanted false positives.

Despite not having the same final purpose of cervical cell abnormality identification, van der Laak [66] also presents pertinent work for adequacy assessment. Discriminant functions (DF) classifiers are proposed in order to recognize debris and inflammatory cells as well as distinguish nuclei from different types. Specifically, nuclei compactness and maximum radius were identified as the best features to discriminate inflammatory cells.

On the other hand, the emergence of automated technologies for cervical cancer screening have been demonstrated to reduce unsatisfactory slide rates [44,45,67], suggesting that computerized solutions may be able to screen and diagnose slides with lower cellularity thresholds or with the presence of artifacts. In fact, although computer algorithms try to mimic human perception, decision making processes can obviously be different, and therefore adequacy criteria should be adapted to the particularities of each specific algorithm.

### 4.3. Segmentation

The first step in cytology diagnosis is the correct identification of cells and respective structural constituents. Since most abnormalities used as guidelines to cervical cancer diagnosis are related to the morphology of the nucleus and cytoplasm, an accurate segmentation is an essential prerequisite for screening solutions.

Historically, the first solutions to the segmentation problem only dealt with clear free-lying cells. Overall, pipelines included three main steps: background extraction; cell localization; and cell boundary determination. Most approaches to do so were mainly based on simple image histogram thresholding methods preceded by basic mean filters for noise removal. Optical density information [68], gradient and compactness information [69], grey level brightness [70,71] or energy [70] are some of the information extracted to build the histograms. The most inherent difficulty was in finding the optimal threshold [71,72]. These works demonstrated remarkable performance for the images under study but failed with more complex cases, so direct application to Pap smears was not possible. Following research evolved around several techniques, which will be the major focus of this section. The reviewed works encompass segmentation of both single and overlapping cells, with the most relevant approaches being summarized in Table 3.

**Mathematical morphology** based methods exploiting color information are also consistently cited, especially by using multi-scale watershed segmentation algorithms. Earlier works with this methodology were only able to detect cell locations and not exactly segment the nuclei [94]. Later approaches improved the detection phase, particularly by coupling a clustering step with supervised (SVM) and unsupervised (K-means) techniques, in order to classify each region and remove false positives [65,74,95]. In the work of Genctav et al. [74], a multi-scale watershed algorithm is first employed to hierarchically segment cell regions in a parametric-free way, followed by the binary classification of the nuclei and cytoplasm based on multiple spectral and shape features.

**Edge detection** algorithms removed any requirement for prior knowledge of the image, although entailing better pre-processing methods than simple thresholding techniques for noise removal and contrast enhancement. Among them, bi-group enhancers have been used in order to better discriminate pixels near object contours, with the goal of emphasizing and isolating those regions. Particularly, curves on an image can be detected after thresholding the image gradient obtained through a canny edge detector algorithm, an approach that can be improved by coupling other techniques like morphological processes [96], color clustering [97], Sobel operators, or non-maximum suppression [98,99]. All these referred techniques are simple and effective measures that do not require considerable computational effort. However, when dealing with complex data containing intensity variations, poor contrast, artifacts, or overlapping cells, they are not suitable to be used individually, thus its implementation is mostly done as sub-steps within a framework. Thus, more sophisticated methods with combinations of these methods and other optimization algorithms were later introduced.

**Active contours**, or **Snakes** (2D), also known as deformable models for its 3D version, have been widely proposed for cervical cell segmentation, especially when nuclei borders are not clearly identified in the sample images [100]. Nuclei segmentation was addressed by Bamford et al. [76] in a Viterbi search-based dual active contour algorithm. A study by Plissiti et al. [101] also introduced a deformable model driven by physical models, in order to improve the detection performance and address possible nuclei overlapping cases. Also, the Gradient Vector Flow (GVF) deformable model was incorporated for the final estimation of cell nuclei [102], and cell nuclei and cytoplasm [77,103]. Finally, in a study by Harandi et al. [104], an automatic method of geometric active contour without re-initialization was implemented with ThinPrep images, where cell localization was performed in low resolution with cell structural part segmentation in high resolution.

**Pixel classification** schemes have been said to avoid the oversegmentation of the watershed algorithm [105]. Implemented approaches include pixel classification methods based on K-means and Bayesian classifiers [106], local and global statistical likelihood criterion [107], fuzzy-based techniques [108], and modified seed-based region growing [109]. A multifractal algorithm followed by a genetic algorithm optimization was also used in a study by Lassouaoui and Hamani [110]. Considering the nuclei ellipse-based shape, some approaches used parametric [72] or elliptical models [111] in a **template matching** manner. A more robust method was proposed in a study by Chen et al. [100], in which segmentation was achieved by finding the most similar model from a set of examples with different shapes and textures in a supervised learning manner. Despite achieving a good performance, these methods were only used to detect the cell’s nuclei. Differently, Nosrati et al. [80] suggested that elliptical shapes do not accurately model cervical cells, using a star-shape prior instead. By coupling it with local thresholding, a circle Hough Transform and a RF classifier, the latter was able to address both nuclei and cytoplasm detection on overlapping cells. Further work on overlapping cells images combined a circular shape function to increase the robustness of a fuzzy c-means clustering algorithm [112].

Additionally, **graph-based** methods have also had some attention on this topic. Within these, active contours are commonly used to map image parts into a graph, whose best graph-cut will represent the best segmentation, iteratively optimized through dynamic programming [113,114]. Other implementation proposed two modified variants of Poisson distribution to model the nucleus and the cytoplasm [82], where a local adaptive graph-cut (LAGC) technique was implemented to cope with heterogeneous illumination and non-uniform staining. This technique is also employed by Zhang et al. [54] to refine a previous nucleus segmentation, where four cell region classes (cytoplasm, nucleus, inflammation and debris) are globally discriminated through a graph-cut, followed by a LAGC to refine the segmentation.

More recently, new cervical cells segmentation methods were also proposed, based on **machine learning (ML)** algorithms. In a study by Zhang et al. [54], several ML algorithms were compared for segmentation refinement with an artifact-nucleus classifier, for which the best performed one obtained by a random forest. Supervised and unsupervised methods were jointly used with other robust refinement techniques to classify image patches or superpixels from extracted features. Examples include modifier Adaboost detectors [115], SVM [88] or Gaussian mixture models [116]. Additionally, a novel superpixel-based Markov random field (MRF) segmentation for non-overlapping cells was introduced in a study by Zhao et al. [87]. With superpixels as MRF node-elements, a labeled map was modeled and optimized through a gap-search mechanism, said to be much faster than normal pixel-based and superpixel-based algorithms.

#### Overlapping Cells

Earlier attempts focused on segmenting cellular nuclei of isolated or only partially overlapped cells, a scenario that is not completely realistic. Apart from a large degree of overlapping and poor cytoplasmic boundary contrast in some cases, the complexity of the segmentation task is also increased by the presence of artifacts and the great amount of cell shape variation. Some works addressed the issue only by solving the clustered nuclei case [74,101,117] whereas individual cytoplasm segmentation remained a challenge. More recently, literature on the topic increased exponentially, in part by the release of the ISBI challenges in 2014 and 2015 with public available datasets (see Section 3.3) of multiple overlapping cells. Superpixel or level-set methods are among the most popular solutions presented.

Regarding the first challenge, three solutions [79,81,118] were evaluated in a study by Lu et al. [34], presenting similar results, all superpixel-based and with three main steps: cell clumps segmentation; nuclei detection; and cytoplasm boundaries detection. Nosrati et al. used a combination of maximally stable extremal region (MSER), random forest classifier and active contour algorithm [118]. Their work was later extended with improved results in a study by Nosrati et al. [80]. In a study by Ushizima et al. [81], thresholding techniques estimate a first segmentation, later refined through a combination of graph-based region growing and Voronoi diagrams. Lastly, the original solution proposed by the challenge authors was also presented as a baseline method [33], with an improved optimization energy function from the work in a study by Lu et al. [79]. Unsupervised gaussian mixture models (GMM) divided superpixel maps into background and cell clumps with the nuclei then being detected with a MSER algorithm, after which several level set functions constrained by shape priors and other geometric and color features assign each one to a cytoplasm region.

For the second challenge, solutions made use of the multiple focal-plane images provided in the 2015 dataset. Similarly to 2014’s works, Phoulady et al. [83] solved the cell clump and nuclei segmentation by an iterative thresholding allied with a learned GMM based on pixel intensities. Further cytoplasm boundary detection was carried out on different focal images, previously divided into a grid based on edge strength. After the release of the results, Phoulady’s group has proposed several improvements with increasingly better results [36,84,85]. In their most recent work, the group proposed the use of a convolutional neural network (CNN) to classify image patches, proven to achieve a superior and more generalized solution when compared to previous state of the art methods [36]. The second winning algorithm [119] was an improved version of the first challenge solution [81] enhanced to work with multi-layer volumes. The superpixel map with the Voronoi diagram was used as an initial estimation, followed by a combination of the extracted contours of each of the focals and the contours given by a Canny edge detector on the extended depth field (EDF) image. It should be noted that the previous works served as a good benchmark for posterior publications on the topic.

Several works have claimed to achieve similar or better quantitative and qualitative performance with different proposed techniques, including multi-pass fast watershed algorithms [75], fragment-based graphs (rather than pixel based) or simple linear iterative clustering and shape prior based algorithms [86,89,120,121,122]. Multi-level set algorithms such as texture pattern-based with optimized integrated feature vectors [123] or with distance regularized evolution [124] have also been popular approaches. As far as ML algorithms are concerned, examples include employing an SVM with superpixel-based features to discretize structural cell components [86].

Furthermore, increasing computational power led to research works on **deep learning** approaches. Although more resource-hungry and less interpretable (which can be an essential factor for healthcare applications), these are often much more automatic and can drop complex pre-processing and computer vision methods that can sometimes be quite erratic and with variable performance. In a study by Braz et al. [125], an approach based on CNNs was proposed to automatically detect the nuclei of both free-lying and overlapping cervical cells. The network was fed with image patches and trained to classify its central pixels into background, cytoplasm and nucleus, achieving similar performance to more classical state-of-art methodologies. Additionally, a patch-based CNN classification with selective pre-processing was implemented in a study by Gautam et al. [92]. It should be noted that the authors argued that no image pre-processing was required in more heterogeneous nuclei, but image contrast should be enhanced if the nuclei presented more homogeneous characteristics. Thus, two different CNN architectures, created by the Visual Geometry Group from the University of Oxford (VGGNet), were trained from scratch, using pre-processed or non-pre-processed cell images, according to nucleus homogeneity. Similar CNN-based approaches were followed in [36,89,90,93] stating an increase in the segmentation performance.

### 4.4. Classification

The common pipeline for cervical cell automated analysis usually includes a cell segmentation step, followed by an abnormality classification step. Feature-based machine learning algorithms are frequently used for classification purposes, and more recently, deep learning approaches. It should be noted that as there are different types of cells to be classified, different binary and multi-class approaches were already proposed in the literature. Additionally, cytology results obtained in clinical practice take into consideration not only image analysis but also clinical information, and consequently some exploratory research regarding multimodal classification is also already available.

Taking into account this wide range of possibilities, this section aims to provide a detailed review of the most relevant classification approaches for cervical cells already proposed in the literature, being the respective summary presented in Table 4.

#### 4.4.1. Feature-Based Classification

This type of classification is based on feature extraction, a process that aims to improve computer vision tasks by reducing the computational complexity of subsequent processes, as well as improving the recognition performance on unknown novel data. Since the in-depth understanding of the domain-specific knowledge gained by human experts on the problem being addressed can be of extreme importance for the design of a reliable and effective feature extraction engine [139], we present a set of popular image features already in use to characterize cervical cells.

#### Cellular Features

The number of extracted image features vary according to the algorithm or complexity required, and are chosen regarding their discriminative power for classification purposes. Popular features in the literature include size, shape, color and textural characteristics related to malignant associated changes cited in TBS [26] and briefly described in Section 3.2.

Table 5 summarizes some commonly used features. Among them, nuclei and cytoplasm (N/C) ratio, nuclei and cytoplasm brightness and nuclei area can be highlighted as some of the most discriminative, while contextual information is introduced to improve specificity [54]. Some other works discuss the usefulness and importance of the cited features in the recognizing of cervical cancer pathological cases. Even though both nucleus and cytoplasm characteristics seem to be considered useful [32,74], some recent literature has argued that nuclei features have a higher discriminative ability [78]. As far as feature types are concerned, Bengtsson and Malm [48] consider chromatin patterns and texture to be the most informative. Lorenzo-Ginori et al. [140] demonstrated better performance when adding texture information rather than using morphology features only. Color information was also noted to provide extra useful information [141].

#### Classification Algorithms

First attempts to classify cervical cancer cytological images were based on Bayesian binary classifiers [144,145] but were limited to more simplistic data without too much variation. More complex approaches were latter introduced, using different ML algorithms.

**Support vector machine** is probably one of the most cited techniques in the literature. In a study by Huang et al. and Cheng et al. [126,146], the authors used SVMs with several filter and wrapper feature selection methods applied to cervical cells. The work by Mariarputham et al. [127] compared the usage of different types of kernels, including linear, quadratic and a radial basis function (RBF) kernels. Although with similar statistical results, the linear kernel had the best performance. By evaluating the SVM classification with seven different sets of texture features, the authors also concluded that the optimal feature set is dependent on the cervical cancer stage. A study by Zhao et al. [128] presents a RBF-SVM that automatically classifies smaller image blocks into one of six types: background; blocks with few white cells; blocks with many white cells; blocks with clustered white cells; blocks with normal epithelial cells; and blocks with suspicious epithelial cells. The work stated that texture and colour histogram features are significantly different in blocks with suspicious cells. In this way, the SVM was able to classify block features, avoiding the segmentation step, and save a lot of computational time. Despite presenting promising results, the evaluation was performed on a small number of images, so further validation should be done. Additionally, avoiding conventional segmentation using CNN for feature extraction also produced good results in a study by Hyeon et al. [147], where a least square support vector machine (LSSVM) outperformed a softmax regression. An RBF-SVM also obtained the best results in a study by Bora et al. [148], outperforming logistic regression, random forest and Adaboost.

**Artificial neural networks (ANN)** is also one of the most widespread classification techniques. In a study by Mango [149], two separate feed-forward neural networks were implemented for independent processing of single-cell and cell-cluster images, outputting an abnormality score. Several neural network (NN) architectures were implemented for this purpose, such as adaptive resonance theory (ART) based [71], RBF [71,150], neural network-relevance vector machine [123], as well as the most common multilayer perception (MLP) [54,151,152], including its hybrid form (HMLP) [153,154]. The latter have also been enhanced in a hierarchical way by having a double HMLP, one for normal/abnormal cell classification and the other to classify the abnormal samples into HSIL or LSIL [129]. Other works combined ANN with the use of fuzzy based techniques. In a study by Kim et al. [71], a fuzzy c-means algorithm was associated to generate the network’s middle layer. Fuzzy logic rules were firstly applied in a study by Li and Najarian [155], followed by a multilayer sigmoid neural network for the cases where the fuzzy classification was unclear. In a study by Gupta et al. [156], the authors demonstrated the effectiveness of ANN for cervical cell classification by comparing it with several other classifiers. Among 15 algorithms, including naïve Bayes, decision trees, random forest, bagging algorithms or RBF neural networks (RBF-NN), a backpropagation-based MLP was the best performed one in both binary (two classes) and multi-class (seven classes) versions.

**Unsupervised classifiers** group data given similarity measures, unlike the previously described supervised methods that require labelled images for training. Collecting sufficient and balanced number of samples representative of all the classes is usually a very challenging task, particularly in clinical scenarios where healthy data are usually much more abundant. This creates unbalanced data which may lead to biased classifiers. Besides that, the process of examining and labelling data is tedious, requiring a lot of time from specialists in order to build a consistent dataset with enough variability. To overcome these issues, some unsupervised techniques have been proposed. One of the most pertinent works within cervical cancer classification automatically ranks cells according to their abnormality degrees without any need for parameters adjustment [74]. A binary tree is constructed using hierarchical clustering from extracted nucleus and cytoplasm features, where each cell composes an individual leaf (cluster). Subsequent levels are formed by merging the two most similar clusters, where the ranking is obtained by computing the optimal leaf ordering. Another example is presented in a study by Plissiti et al. [78], where a fuzzy c-means algorithm outperformed spectral clustering for cervical cells classification solely based on nucleus features.

Other supervised methods have been used, such as **k-Nearest Neighbours algorithms (k-NN)** [132,133]. The authors in a study by Marinakis et al. [131] integrated fuzzy intuition into the k-NN’s membership values and by combining it with a quantum-behaved particle swarm optimization for feature selection, it outperformed SVM, naïve Bayes and RF approaches. Multi-classifiers were also proposed through **Ensemble algorithms** like in a study by Bora et al. [134], where final classification was obtained after a weighted majority voting of a LSSVM, MLP and RF classifiers. In this particular work, each classifier obtained good performance individually, but the best performance was achieved by the ensemble approach. The same was verified in a study by Gomez et al. [135] with the usage of two ensemble approaches (Bagging + MLP and AdaBoost + Logistic Model Tree), as well as in a study by Sarwar et al. [157], where the predictive performance of 15 different algorithms was improved by merging them through a hybrid ensemble technique.

Despite the impressive results reported by these different classification methodologies, it should be noted that the performance is highly dependent on the used segmentation technique, parameter optimization, dataset used, extracted features or problem dimension, and complexity. Since choosing the best classifier algorithm can be quite subjective, a few authors have already addressed this ambiguity and tried to make a proper and fair comparison between different approaches. Morphometric and texture features were fed to a Linear classifier, a k-NN, a Mahalanobis distance classifier and an SVM [140]. Among them, the SVM with a Gaussian RBF kernel achieved the best performance, closely followed by the Mahalanobis classifier. Additionally, in a study by Chankong et al. [130] five classifiers types were extensively analyzed on three distinct datasets. Both binary and multi-class problems were investigated, as well as the usage of a varying number of features. Within a Bayesian classifier, a linear discriminant analysis algorithm, a k-NN (k = 7), a three-layer backpropagation ANN and a RBF-SVM, the usage of the ANN using nine features stood out as the best option for the binary and the multi-class problem. Finally, contrary to Chankong et al. study [130] whose images are single-cell only, the work by Zhang et al. [54] studied the complete workflow from acquisition to classification of multi-cell images. A MLP, an AdaBoost, a RF, and an SVM are applied to different purposes, differentiate nuclei from artifacts, and classify normal/abnormal nuclei (binary problem). For the first case, RF was the best performing algorithm whereas MLP was chosen for the latter case.

#### 4.4.2. Deep Learning Classification

Most of the traditional approaches are limited by the vast variability of cell appearance. Furthermore, extraction and selection of specific hand-craft features may ignore important or more complex discriminative information for abnormality detection. Conversely, deep learning approaches in the form of a convolutional neural network (CNN) automatically extract deep hierarchical features, not requiring previous segmentation steps. (Although ANN can also be referred to as deep learning due to a possible deep architecture (high number of hidden layers), we consider deep learning as the segmentation-free methods whose inputs are image pixels directly, and not numerical data previous extracted.) Aa study by Rasche et al. [141] verified that a Deep Belief Network achieved higher accuracy when compared to a traditional methodology with segmentation and feature extraction followed by an SVM in different classification tasks. However, some issues in discriminating subtle structural differences were identified, particularly between LSIL and healthy types.

On the other hand, the learning process of these networks not only requires much more computational time and power but also large labeled datasets. Specifically in terms of cervical cells, publicly available annotated data is very limited, and consequently some works on cervical cell classification have implemented CNN architectures through transfer learning techniques. These approaches use a pre-trained network as a base model on extensive image datasets (e.g., ImageNet [158]), exploiting the more generic features learned in the first layers, and focusing on fine-tuning the last task-specific layers using target data. As an example, in a study by Zhang et al. [136] a segmentation-free approach was implemented by fine-tuning an AlexNet on two Pap smear datasets to classify nuclei-centered image patches. Due to the lack of data available and to increase its variability, data augmentation and balancing techniques were also employed. By doing so, the network becomes more robust, less biased, as well as rotation and scale invariant, increasing its performance. Despite surpassing previous classical state of the art algorithms, the method is limited by its run time (around 3.5 s per input, which is unfeasible if we consider that a single slide may contain roughly 300,000 cells).

In a following work, Jith et al. [137] proposed a simpler and smaller architecture, with only three of the AlexNet’s convolutional layers, achieving a more feasible implementation with reduced computational complexity and similar accuracies. The same transfer learning technique was further tested with other CNN model architectures by Lin et al. [138], including AlexNet, GoogleNet, ResNet and DenseNet. The GoogleNet outperformed all the others on both 2-class and 7-class classification. The authors also proposed combining the raw Red-Blue-Green data (three channels) of the image patches with the ground truth segmentation masks of nuclei and cytoplasm (two additional channels), creating a five-dimensional input. All the networks achieved better performance with the five-channel input, although results were only marginally superior.

Given its simpler architecture, an AlexNet was also chosen in a study by Gautam et al. [91] and compared with other methods. It achieved the best accuracy in a 2-class problem, whereas in a 7-class was slightly outperformed by an ANN [130]. Considering the underperformance in the 7-class problem, a decision tree-based classification using Transfer Learning was used as an alternative. The algorithm iteratively classifies samples at each stage, starting with a normal/abnormal classifier and going until the last stage where it distinguishes between the two highest levels of abnormality. Each node of the tree is composed of an AlexNet CNN pre-trained on ImageNet. This procedure is proven to be more accurate than multiclass classification on a single AlexNet. Additionally, this work also investigated if sophisticated segmentation was necessary in the presence of multi-cell images. Easier cell-nuclei detection was shown to be more effective than an accurate segmentation for CNN-based classification.

In addition to having demonstrated very good quantitative results, most of the reviewed approaches still do not address the topics of interpretability and explainability. The lack of transparency in these models might pose serious restrictions on its future use in clinical practice, since they need to inspire confidence and promote acceptance over the medical community, but also be compliant with certification procedures. One particular methodology [159] started to address these topics through the usage of patient clinical information, in which a deep variational autoencoder was introduced within a neural network to assess the risk of cervical cancer. Particularly, the loss function was constructed to minimize the trade-off between data reconstruction and classification performance, being possible to partially study the impact of each feature and find correlations among the decision process. It is also worth mentioning that recent image-based techniques for CNNs, like network filter visualization or activation maps generation, have already been explored in different medical areas to mitigate the lack of model transparency. However, the applicability and relevance of the aforementioned techniques for cervical cell analysis still lies unexplored.

In sum, recent deep learning-based classifications have demonstrated promising results when compared to previous classical approaches, especially considering that they may learn to cope much better with image and cell structure variability. However, the current usage in real CADx systems still faces several challenges, not only because these are more time and resource hungry approaches, but also because they usually present a significantly greater complexity, being harder to clearly explain the rationale behind the automated decision. Additionally, due to the lack of large and more realistic public datasets, most of these implementations have been applied on single-cell images, requiring previous cell detection and extraction. Future work might focus on smear-level classification rather than single-cell.

#### 4.4.3. Binary vs. Multi-Class

Most works mentioned a better performance for the 2-class problem when compared to the multi-class one (3, 5 or 7 classes) [126,130,133,138,160]. In order to accurately learn the particularities of each class in an unbiased way, a meaningful quantity of data equally distributed is required, which is in most of the cases difficult to acquire. As an example, in the Herlev dataset, the normal columnar class was often indicated as less sensitive and misclassified as severe dysplasia cells, probably due to similar characteristics in morphology such as dark nuclei and small-size cytoplasm [32,138]. Rasche et al. [141] also suggested building more specific feature extraction processes or even separate classifiers to improve discrimination between LSIL and healthy cell types, whose subtle structural differences seem to be a source of confusion. This might explain the performance gap reported in initial works between binary and multi-class problems, although more recently some studies contributed with significant improvements and brought the latter close to binary levels of performance.

#### 4.4.4. Multimodal Classification

Cytological image classification is a crucial step in an automated cervical cancer diagnosis system. However, errors and uncertainties introduced in the several necessary steps may decrease the diagnosis accuracy. In addition, due to different risk factors and/or patient clinical history, what may be worthy of attention for one particular patient, can be completely normal for another patient. Fusing multimodal information, for instance textual and image data, can potentially improve the diagnosis performance. In particular, identification at an early stage of women with LSIL lesions likely to progress has been identified as one of the main advantages of a multimodal screening [161]. Another great advantage for cytology applications is the reduced number of required analysed cells and the number of necessary biopsies for cancer diagnosis confirmation [162].

Specifically for cervical cancer diagnosis, considering the usage of different diagnostic tests in the standard workflows that output different types of information, as well as the existence of correlations between several risk factors, some ML frameworks already explored the combination of various adjuvant screening methods, for instance merging image data from cervigrams with textual/numeric data of high-level information from medical records (e.g., age, HPV status, Pap Test results, etc.). Integration of multimodal information is generally done at the final stage, and for this reason they are also referred as late fusion methods. In a study by Xu et al. [163], two SVMs were trained separately, one for image data and other for non-image data, after which, thresholding on its weighted sum outputs the final decision. Alternatively, in a study by Song et al. [164] the adoption of information gain and gradient-based approaches to automatically learn the relative weights of different tests was proposed. Data similarity from different modalities is aggregated and compared to the training set to find the final decision. On the other hand, these methods usually analyze clinical variables separately, without considering useful information provided by their correlations. Thus, other implementations use an early fusion methodology instead. Such is the case of a study by Xu et al. [165], where deep image features are extracted from a fine-tuned AlexNet and compressed to a 13-dimensional vector in order to meet the dimension of the non-image data. After this, a number of joint connected layers are used to simultaneously concatenate all the information and learn its correlations. The networks learning procedure also enables backpropagation to the CNN layers such that the previous CNN can extract visual features that better complement the clinical information. Another interesting approach is suggested by Fernandes et al. [37], in which a partial transfer framework predicted a patient’s risk when multiple screening strategies were available (numeric and textual). Built on the premise that individual models for related tasks should share some high-level proprieties, they explore regularization techniques to transfer contributions of each individual feature into common linear models.

Considering exclusively cytological data, the application of different stains on the same sample may provide additional information not accessible through single-staining images. A multimodal cell analysis algorithm has been proposed in a study by Ropers et al. [166] for matching identical cells in different stainings, whose information can later be merged. In another work, Bell et al. [167] also evaluated an acquisition procedure that can automatically relocate and autofocus cells after an image has been acquired in a first stain.

Another topic that is highly relevant for multimodal classification is methods for missing data handling. When aggregating different clinical test results and clinical information for the same clinical case, it is quite common that not all the expected data is available. Several works (not specifically applied for the cervical scenario) have already explored approaches to tackle this problem. In a study by Xu et al. [165], the authors complete the missing values by averaging the respective dimension on training data, while in a study from 2015 they [163] present two algorithms: image classifier supervised mean imputation, and image classifier supervised linear interpolation for missing image data. Alternatively, in a study by Ngiam et al. [168] a deep autoencoder learned to reconstruct data when missing, following a similar encoder-decoder approach by Cai et al. [169], whose model is able to generate high-quality missing modality images.

## 5. Discussion

Objective comparison between different works in the literature is a difficult task. Commonly, widely distinct evaluation methodologies are used, including different datasets (often privately acquired), tests performed, and selected performance metrics. Furthermore, robust datasets that can be adopted in every step of a complete automatic pipeline (including pre-processing, segmentation and classification) were not available until the release of the CERVIX93 dataset in November, 2018. In fact, the Herlev dataset has different cells types and ground truth labels, but its images do not have realistic issues like overlapping cells, inflammatory cells or artifacts. On the other hand, the two ISBI challenge datasets data only provided information on cells segmentation, lacking more variable cell types and respective abnormalities classification. As follows, in this section we perform a practical critical appreciation, with a major focus on works tested on publicly available data and with clear evaluation methodologies. An overall summary is also schematized in Table 3 and Table 4 for segmentation and classification, respectively.

### 5.1. Segmentation

In terms of cell segmentation, there is an obvious division between methodologies before and after the ISBI challenge datasets release. Pre-challenge, most of the literature is tested on private datasets or on the Herlev dataset. Here, the works of Genctav et al. [74] and Li et al. [77] can be highlighted. They implement different approaches to the problem, unsupervised and supervised respectively, both achieving similar performances and being among the most cited methodologies. In addition to the Herlev dataset, the Genctav et al. dataset [74] is also evaluated on a more realist private collected set of samples, with overlapping cells and presence of artifacts, verifying its robustness to image variability. Even though they were released pre-challenge, these approaches were later reproduced and compared in more recent works, serving as the baseline methods for many other implementations.

Among the after-ISBI works, we refer to a study by Lu et al. [34]. It compares and evaluates quantitatively and qualitatively the three successful submissions for the 2014 challenge, whose overall winner was Ushizima et al. [81]. Nevertheless, Lu et al. [33] (the baseline method) and Nostari et al. [118] (later enhanced in [80]) also present very good alternatives and even obtained better performance in some metrics. For instance, Lu et al. [33] has the best cytoplasm segmentation performance regarding true positive rate, but it also produces the highest object-based false negative rate. In this way, the referred paper offers a good analysis to the pros and cons of some of the best segmentation techniques for cervical cells. This is an important contribution and can serve as a baseline for designing an automated intelligent system, as this process will always involve weighting between different metrics, such as time and computational complexity, sensitivity, specificity, etc. The usage of accuracy as the unique considered performance metric will certainly not be enough to properly evaluate the potential of a CADx system.

Furthermore, Phoulady et al. [85] and Ramalho et al. [119] (the extension of Ushizima et al. [81]) pose as the best approaches when using the EDF images from the ISBI15 dataset. Finally, the work of Phoulady et al. [85] on EDF samples was enhanced after the release of the CERVIX93 dataset through the employment of a CNN for nuclei segmentation [36], outperforming the previous presented works [81,85]. It should be noted that, although presenting very good results on ISBI datasets, performance of other state of art methodologies deteriorates when applied to more challenging images such as CERVIX93 images. As a disadvantage, the work Phoulady and Mouton [36] entails more computational effort, which can be a limitation for low-cost application settings, and only provides a methodology for nuclei detection.

### 5.2. Classification

As far as the classification step, the benchmark used as a baseline in all the literature is a study by Jantzen et al. [32], with the release of the Herlev dataset. Generally, SVMs and ANNs are ranked best within this set of samples. Though, this goes back to 2005, so their performance is certainly not up to date. Particularly, the multi-class classification problem (7-class) had significantly lower performance until more recently developments. Difficulties in distinguishing subtle differences between classes are often mentioned. Furthermore, Herlev’s “Normal Columnar Epithelial“ class is also a source of much misclassification, given that, although not having any abnormalities, its cell type and shape is rather different than “Normal Squamous” cells.

While the usage of more granular and detailed classification methods may result in more versatile CADx systems, it should be taken into account that focusing exclusively on complex multiclass approaches may not always be the best option, as it can result in lower performances for particular use cases. Some methodologies may be better for multiclass classification, whereas others may be extremely accurate for binary cases. Thus, the selection of the most suitable approach should always take into consideration the requirements and major objective of the CADx system being designed. Nevertheless, some studies have recently accomplished outstanding performance on both binary and multi-class problems. One of them is a study by Chankong et al. [130] that presents an extensive analysis of both segmentation and classification, comparing their work with the best algorithms in the literature on three different datasets. Assessment of several classifiers and respective feature dimensionality is also made, providing various performance metrics. Their segmentation results are in line with most relevant works, although not outperforming the results on Genctav [74] and Li [77].

As far as deep learning approaches are concerned, we highlight two works. The work of Zhang et al. [136] was the first major CNN-based approach proposed with results that are in line with a study by Chankong et al. [130], although marginally inferior. Compared to feature-based approaches, it does not require extensive pre-processing nor segmentation, being able to capture inherent but essential features. Furthermore, since features and rules are not “hard-coded”, it has the potential to be more robust to noise, as well as more generalizable to other datasets. Despite these advantages, the method is extremely slow, taking 3.5 seconds per image patch, which makes it unfeasible for clinical settings. In a study by Jith et al. [137], is explored a more lightweight version of the aforementioned approach, which even achieves better results. However, the performance was only evaluated in terms of accuracy, thus requiring further assessment to prove its efficiency. The second highlighted deep learning work is by Gautam et al. [91], which provides a comprehensive description of CNN-based algorithms for cervical cancer. Their binary performance exceeds results of Zhang et al. and Chankong et al [130,136], whereas the 7-class problem is similar to [130]. However, once again the only compared metric is accuracy. Nevertheless, they present an alternative implementation of Zhang [136], analyzing several CNN-based classifier hypotheses and further assessing the importance of the cell segmentation on this type of approach.

In addition, the work of Bora et al. [134] should also be mentioned, which is one of the few studies that investigates not only cell-level but also smear-level classification. The authors demonstrate the perks of combining different algorithms, evaluating their single versions and an ensemble, obtaining very good results on Herlev’s images along with two other generated datasets, very similar to those in a study by Gautam et al. [91] and Chankong et al. [130]. An in-depth analysis on various features subsets is done, verifying the importance of shape, texture and color features for cervical cancer diagnosis.

## 6. Conclusions and Considerations for Next Generation of CADx Systems

This review offers a contextualization of the current cervical cancer screening procedures, as well as an in-depth analysis of the most relevant computational methods available on the literature for cervical cells analysis. Overall, a wide range of methodologies already proposed for segmentation and classification purposes were reviewed and compared, each with different advantages and disadvantages as detailed in Section 5. Since the major goal of this timely review is to support the development of new automated tools that can facilitate the cervical screening procedures, we provide some considerations regarding the next generation of computer aided diagnosis systems and future research directions. Particularly, we will focus on topics related with adequacy assessment, segmentation and classification.

### 6.1. Adequacy Assessment

Although being essential to the screening process, smear adequacy assessment is still a topic scarcely addressed in the literature. Most works simply ignore it, while others present some work in the detection and removal of unwanted objects such as inflammatory cells, dirt, blood or other artifacts. Here, we point essentially to a study by Malm et al. [63] for a complete analysis and implementation on Pap smears debris removal and to Zhang et al. [54] who builds an iterative artifact filter. Despite some efforts on the topic, this is an inherent difficult task, and completely acceptable solutions are still yet to be found.

It is also worth noting that one of necessary conditions for smear adequacy defined by the Bethesda system is the minimum cellularity, but this is rarely mentioned on computerized solutions research. This requirement entails differentiation between squamous and glandular cells, given that satisfactory cellularity only takes into account the squamous type. However, work on glandular cells detection and classification is very scarce. Even the commercially available systems have reported high false-negative rates on this type of cells, with low specificity and sensitive rates [170]. The Bethesda system emphasizes that *“cervical cytology is primarily a screening test for squamous lesions; it is not intended to screen for endometrial lesions and should not be used to evaluate suspected endometrial abnormalities. (…) it is not feasible for a screening test to detect every malignancy”* [26]. In fact, finding squamous abnormalities is 10 times more frequent than glandular atypia in cervical cytology. On the other hand, of those reported findings, a considerable amount was later diagnosed with significant lesions (9%–38%) or invasive carcinoma (3%–17%) in follow-up tests [171]. Considering this, although not being one of the current major priorities in terms of CADx systems, some effort should also be put into detecting glandular cellular abnormalities, not only to determine satisfactory/unsatisfactory slides, but also because it can be evidence of more serious complications.

### 6.2. Segmentation

While segmentation is one the crucial steps of the traditional workflow of image-based diagnosis systems, it is often one of its computational time bottlenecks. Specific segmentation, robust to many variations and extra factors, requires complex computer vision algorithms. It may be insignificant on an image-level but considering that one simple cytology test involves thousands of cell analysis, it can quickly become unfeasible. Segmentation-free approaches like CNNs can release the burden of precise segmentation process, but they will also probably increase computational efforts on the classification part. Block-processing methodologies like Zhao et al. [128] and Zhang et al. [172], as well as MAC approaches (instead of analysis of all the smear cells) seem a good lightweight alternative that avoid the dependence on accurate segmentation. However, these approaches can be less controlled, and consequently less transparent regarding the rationale behind the automated decision.

Considerations on how accurate and precise the segmentation should be are also pertinent. Is it enough to extract information on the nuclei? Some of the approaches could successfully classify cells based on nucleus features only, but the best performing methodologies involve feature extraction from the entire cell. Either way, is it enough to have a more flexible segmentation? Can it help to increase not only the sensitivity process, but also the medical community acceptance to computerized solutions? Currently there are no obvious or correct answers, but the selection of the most suitable approach should always take into consideration the requirements and major objective of the CADx system being designed.

### 6.3. Classification

Algorithms parametrization will affect the final system outcome, and are usually calibrated to have the best trade-off between sensitivity and specificity, according to the system’s goals and requirements. For instance, most nuclei segmentation algorithms will certainly produce false positive candidates, for instance, some artifacts that may look like a nucleus. Finding appropriate parameter values that will report very few false positives (FP), but simultaneously will not systematically exclude abnormal cells (i.e., deliver very few false negatives) is a challenging and delicate task. The most appropriate way of defining these values is looking into cell and debris distribution [60] or employing MAC approaches, but it is important to leave space for some uncertainty in order to assure robustness to other types of “unseen” data.

Globally, in medical applications it is usually desirable not to miss-classify any abnormal case, i.e., zero false negatives. This comes at a cost of an increasing rate of FP, but such might not be the case for cytology systems. It might seem counter-intuitive, but a simple slide can contain several thousands of cells (depending on its preparation) so even a small percentage of FP will make the system almost useless. Hypothetically, if we consider a healthy sample with 20 000 cells and a FP error of 1%, this will result in 200 cells being characterized as abnormal on a supposedly normal slide. On the contrary, assuming an average of 20 abnormal cells in a test slide with diagnosis >= ASC-US [173], with a FN error rate of 20%, we would still be able to detect 16 of the abnormalities if we scan the whole slide. Still, finding an abnormal cell in an abnormal slide is a “needle in a haystack” problem, and the risks of miss-classifying an abnormal as a normal smear are way higher than miss-classifying a normal cell as abnormal, thus sensitivity should not be understated.

To avoid this burden, instead of discrete classification, other more sophisticated approaches are also possible like ranking samples according to their abnormality and priority or extracting the classification output confidence. In this way, most of cells could still be dismissed as “confidently” normal and most of the visual screening workload be removed. In sum, this trade-off should be carefully analyzed and taken into consideration when defining algorithms thresholds and requirements, in order to have balanced CADx systems for cervical cancer screening that confidently meet its major goals.

For final remarks, we identify two future research directions that will likely have a great impact in the development of the next generation of CADx systems for cervical cancer screening and diagnosis. Considering that different diagnostic tests are currently used in standard workflows for cervical cancer diagnosis (which output different types of information), coupled to the existence of strong correlations between several risk factors and/or a patient’s clinical history, allow us to conclude that the potential of multimodal classification approaches cannot be overstated. Automated systems will surely benefit from the fusion of these different streams of information, and current approaches will certainly be outperformed.

Secondly, an increasing demand on artificial intelligence approaches that intrinsically address the topics of interpretability and explainability. The lack of transparency in these models might pose serious restrictions on its future use in clinical practice, since they need to inspire confidence and promote acceptance over the medical community, but also be compliant with certification procedures. Addressing these topics will not only provide better insights about the decision rationale behind ML algorithms (avoiding the concept of “black boxes”), but will also help in closing the gap between engineers and the medical community, thus benefiting patients and society as a whole.

## Figures and Tables

**Table 1:** Summary information about the main classification systems for cervical cancer.

Classification System	Author	Grading Criteria	Reporting Purpose	Clinical Purpose
The Bethesda System (TBS) [26]	United States National Cancer Institute (NCI)	For cervical cytological report (results of microscopic examination of a smear)	Depending on the cells’ extent of abnormality	Screening (test for detecting early changes of the cells of the cervix)
Cervical Intraepithelial Neoplasia (CIN) [27]	Richart R.M.	For histological report (results of microscopic examination of tissue samples)	According to the thickness of the abnormal epithelium	Diagnosis (medical test to aid in the diagnosis or detection of cervical cancer)
TNM [8]	Union for International Cancer Control (UICC)	To document prognostic factors: tumour’s size (T), affected lymph nodes (N) and distant metastases (M)	Based either on clinical description or pathological classification	Staging and tumour risk assessment
FIGO [8]	International Federation of Gynaecology and Obstetrics (FIGO)	To determine the extent of the cervical invasion	Based on clinical examination	Staging and tumour risk assessment

**Table 2:** Public datasets summary. Seg. (Segmentation); Class. (Classification).

Dataset	Year	Type	No Images	Purpose	Description
Herlev [32]	2005	Image	917	Seg. Class.	Single-cell images with segmentation ground-truth. Classification divided in seven classes (Figure 4).
ISBI14 [33,34]	2014	Image	16 EDF + 945 Synthetic	Seg.	Extended depth field (EDF) [35] and synthetic images containing cells with different overlapping degrees. Segmentation of nuclei and cytoplasm (Figure 5).
ISBI15 [33]	2015	Image	17 EDF (each with 20 FOVs)	Seg.	EDF images containing cells with different overlapping degrees and respective fields of view (FOVs). Nuclei and cytoplasm segmentation (Figure 5).
CERVIX93 [36]	2018	Image	93 EDF (each with 20 FOVs)	Seg. Class.	Similiar to ISBI15 images. Classification divided in seven classes (Figure 4). Segmentation only for nuclei points.
Risk-Factors [37,38]	2017	Text	-	Class.	Patient’s information and medical history. Target variables: required diagnosis tests (Hinselmann, Schiller, Cytology and Biopsy). It can be used to infer the patient’s likelihood of having cervical cancer.

**Table 3:** Summary table with highlighted works on cervical cells segmentation. When more than one dataset was used, performance is given only on the public datasets for comparison purposes. Extension works presented by the same author/group are in the same row, with the performance being given for the best case. Acc (Accuracy), Prec (Precision), Rec (Recall), Sp (Specificity), Nuc (Nuclei), Cyt (Cytoplasm), DSC (Dice similarity coefficient) (same as ZSI-Zijdenbos similarity index).

Paper/Authors	Segmentation Technique	Cells Overlap	Datasets	Performance
	*Watersheds*			
Plissiti et al. (2011, 2011) [65,73]	Watershed computation + Refinement based on shape prior. Artifact removal by distance-dependent rule and pixel classification (Fuzzy C-means (FCM), support vector machine (SVM)).	No	Private	FCM: Rec: 90.6% Sp: 75.3%. SVM: Rec: 69.9% Sp: 92.0%
Gençtav et al. 2012 [74]	Multi-scale watershed + Hierarchical unsupervised segmentation tree + Final binary classifier within cell regions	Yes (clumps and nuclei only)	Herlev, Private	(Herlev): Acc: 97%; Prec: 88%. Rec: 93%; DSC: 0.89
Tareef et al. 2018 [75]	Multi-pass watershed + Ellipse fitting	Yes	ISBI 2014, ISBI 2015	(ISBI 2014): Nuc DSC: 0.925; Rec: 95.0%; Prec: 90.6%. (ISBI 2015): Cyt DSC: 0.851
	*Active Contour Models (ACM)/Gradient Vector Flow (GVF)*			
Bamford et al. 1998 [76]	Viterbi search-based dual active contour	No	Private	Acc: 99.6%
Li et al. 2012 [77]	K-means clustering + Edge computation map by Radiating GVF	No	Herlev	DSC: 0.954
Plissiti et al. 2012 [78]	Snake driven by adaptative physical model	Overl. Nuclei	Private	Hausdorf distance: 19.91
	*Level Sets with Shape Priors*			
Lu et al. (2015, 2013) [33,79]	Unsupervised Gaussian mixture models (GMM) + Maximally stable extremal regions (MSER) + Level set with elliptical shape	Yes	ISBI 2014	Nuc Prec:94.2%; Rec:91.2%; DSC:0.921. Cyt DSC: 0.88
Nosrati and Hamarneh 2015 [80]	Random forest (RF) classifier + Level Set with elliptical, 2014, and/or star shape prior, 2015, and Voronoi energy based, 2015	Yes	ISBI 2014	Nuc Prec: 90.1%; Rec:91.6%; DSC:0.900. Cyt DSC: 0.871
	*Graph/Grid-based*			
Ushizima et al. 2015, 3 pages [81]	Graph-based region growing + Voronoi Diagram	Yes	ISBI 2014, ISBI 2015	(ISBI 2014): Nuc Rec: 87.1%; Prec: 96.8%; DSC: 0.914. Cyt DSC:0.872. (ISBI 2015): Cyt DSC: 0.875
Zhang et al. (2014, 2014) [54,82]	Multi-way graph cut globally on the a* channel for background/cell segmentation + Local adaptative graph-cut (LAGC) for nucleus delineation.	Only touching nuclei	Private	Nuc Prec: 85%; Rec: 90%; Cyt Acc: 93%; DSC: 0.93
Phoulady et al. (2015, 2016, 2017) [83,84,85]	Iterative thresholding + GMM Expectation-Maximization (EM) + Grid approach with distance metric from multi-focal images	Yes	ISBI 2014, ISBI 2015	(ISBI 2014): Nuc Prec: 96.1%; Rec: 93.3%. Cyt DSC: 0.901. (ISBI 2015): Cyt DSC: 0.869
	*Machine Learning Classification (Nuclei, Cytoplasm, Background)*			
Tareef et al. 2014 [86]	Linear kernel SVM classifier on superpixels followed by edge enchancement and adaptative thresholding techniques	Yes	ISBI 2014	Nuc Prec: 94.3%; Rec: 92.0%; DSC: 0.926. Cyt: DSC 0.914
Zhao et al. 2016 [87]	Markov random field (MRF) classifier with a Gap-search algorithm + Automatic labeling map	No	Herlev, Private	(Herlev) Nuc DSC: 0.93. Cyt DSC: 0.82
Tareef et al. 2017 [88]	SVM classification + Shape based-guided Level Set based on Sparse Coding for overlapping cytoplasm	Yes	ISBI 2014	Nuc Prec: 95%; Rec: 93%; DSC: 0.93. Cyt DSC: 0.89
	*Convolutional Neural Network (CNN) Segmentation*			
Song et al. (2014, 2017) [89,90]	Multi-scale CNN feature extraction with spatial pyramids + neural network (NN). Refinement: Graph partitioning + Unsupervised Clustering (2015). Dynamic multi-template shape model (2017).	Only touching nuclei (2015). Yes (2017)	Private, ISBI 2015	(ISBI 2015): Nuc DSC: 0.93. Cyt DSC: 0.91
Gautam et al. (2018, 2018) [91,92]	CNN with selective pre-processing based on nucleus size and chromatin pattern + post-processing morphological filtering.	No	Herlev	Prec: 89%; Rec: 91%; DSC:0.90
Tareef et al. 2017 [93]	CNN patch-based for cellular components classification. Cytoplasm estimation by Voronoi Diagram + Level Set with Shape prior	Yes	ISBI 2014	Nuc Prec: 94%; Rec:95%; DSC:0.94.Cyt DSC:0.897

**Table 4:** Summary table with highlighted works on cervical cell classification. When more than one dataset was used, performance is given only on the public datasets for comparison purposes. Extension works presented by the same author/group are in the same line. In this case, performance is given for the best case, which is the most recent work. Acc (Accuracy), Prec (Precision), Rec (Recall), Sp (Specificity), H-mean (Harmonic mean of Sensitivity and Specificity), CCR (Correct Classification Rate), Rs (Spearman rank-order correlation coefficient), k (Cohen’s kappa coefficient), kw (weighted kappa coefficient), RMSE (Root Mean Square Error), OE (Overall Error).

Paper/Authors	Classification Technique	Datasets	Classes	Performance
	*Support Vector Machine (SVM)*			
Chen et al. 2014 [126]	SVM and Fisher linear discriminant classifiers with feature selection filter and wrapper methods. Best: SVM with Recursive Feature Addition (RFA)	Private	2	Acc 98.8%; Rec 91.4%; Sp 99.9%;
Mariarputham et al. 2014 [127]	NN and SVM with different kernels + Feature set (FS). Best: Linear Kernel SVM	Herlev	2, 7 class	Acc: Norm. 96.91%; Interm. 93.89%; Col. 92.35%; Mild 92.33%; Mod. 96.62%; Sev. 92.10%; CIS. 91.72%
Zhao et al. 2016 [128]	Block image partitioning and segmentation. Feature extraction on non-background blocks followed by classification through a radial basis function-SVM.	Private	2-class	Acc 98.98%; Rec 95.0%; Sp 99.33%
	*Artificial Neural Networks (ANN)*			
Mat-Isa et al. 2008 [129]	Cascade Hybrid Multilayer Perceptron (H${}^{2}$MLP). 1st: Abnormal/Normal 2nd: LSIL vs HSIL classifier	Private	3 class	Acc 97.50%; Rec 96.67%; Sp 100%
Chankong et al. 2014 [130]	Extensive comparison of five classifiers and FS. Best: three layer Backpropagation ANN with nine features	Herlev, Private (ERUDIT, LCH)	2, 4, 7 class	(Herlev) 2-class: Acc 99.27%; Rec 99.85%; Sp 96.53%. 7-class: Acc 93.78%; Rec 98.96%; Sp 96.69%;
Zhang et al. 2014 [54]	Artifact classifier + four Iterative Abnormality MLP classifiers	HELBC (Private)	2 class	CCR 94.3%; Rec 88.1%; Sp 100%
	*Unsupervised Classification*			
Marinakis et al. (2006, 2008, 2009) [131,132,133]	K-NN with FS: Tabu Search (2006), Particle Swarm (2008) and Genetic Algorithm (2009)	Herlev, Private	2, 7 class	(Herlev) 2-class: RMSE 0.1796; OE 3.164%. 7-class: RMSE 0.895; OE 4.253%
Gençtav et al. 2012 [74]	Hierarchical clustering tree + optimal leaf ordering that maximizes similarly of adjacent leaves and ranks cells’ abnormality.	Herlev, Hacettepe (Private)	7 class	(Herlev) Rs 0.848; k 0.848; kw 0.848
Plissiti et al. 2012 [78]	Fuzzy C-means and Spectral Clustering based on nuclei features only	Herlev	2, 7 class	FCM H-mean: 90.58%; SClust H-mean: 88.77%
	*Ensemble*			
Bora et al. 2017 [134]	Ensemble of LSSVM, MLP and RF weighted by majority voting. Single cell and smear level classification	Herlev, Private	2, 3 class	(Herlev) 2-class: Acc 96.51%; Rec 98.96%; Sp 89.67%. 3-class: Acc 91.71%; Rec 89.41%; Sp 94.84%;
Gómez et al. 2017 [135]	Comparison of several algorithms. Best: Bagging + MultilayerPerceptron and AdaBoostM1 + LMT	Herlev	2-class	Acc 95.74%
	*Deep Learning*			
Zhang et al. 2017 [136]	Nuclei centered patched-based CNN through Transfer Learning	Herlev, HEMLBC (Private)	2-class:	Acc 98.3%; Rec 98.2%; Sp 98.3%; H-mean 98.3%;
Jith et al. 2018 [137]	CNN based on fine tuned AlexNet	Herlev, Aindra (Private)	2-class	Acc 99.6%
Gautam et al. 2018 [91]	Two patch-based CNNs with selective pre-processing + pre-trained AlexNet classification or Hierarchical Decision Tree with CNN on each leaf	Herlev, Aindra (Private)	2, 7-class	2-class Acc: 99.3%. 7-class Acc: 93.75%
Lin et al. 2019 [138]	Concatenate nucleus centered RGB images patches with cytoplasm and nucleus masks as a five-channel input to several pre-trained CNN	Herlev	2,7-class	2-class: Acc 94.5%; Rec 97.4%; Sp 90.4%. 7-class: Acc 64.5%

**Table 5:** Summary of commonly used image features for cervical cell classification. Some of the features represent more high-level concepts, for its measures and respective extraction we refer to some of its implementations [32,54,61,73,74,140,142,143]. N/C (nucleus/cytoplasm; GLCM (grey-level co-occurrence matrix); SDNRL (standard deviation of the normalized radial length). LBP (local binary pattern). * These characteristics are extracted for both nucleus and cytoplasm.

Shape	Chromatin	Texture	Other
Area *	Brightness *	Multi-nucleus cells	Fourier descriptor
Roundness *	Mean Grey Level	GLCM measures	Nucleus distribution
Longest Diameter *	Intensity Disparity	Optical Density	Nucleus Position
Eccentricity	Minima *	Uniformity	Graph-based (contextual)
Major Axis length	Maxima *	Entropy	
Minor Axis Length	Average Color	Smoothness	
Perimeter *	Boundary intensity	Neighborhood Intensity Disparity	
Elongation *	Smoothness	LBP mean value	
Convexity	Variance	Coarseness	
SDNRL			
N/C ratio			
